# Aspirin inhibits epithelial-to-mesenchymal transition and migration of oncogenic K-ras-expressing non-small cell lung carcinoma cells by down-regulating E-cadherin repressor Slug

**DOI:** 10.1186/s12885-016-2078-7

**Published:** 2016-01-26

**Authors:** Poulami Khan, Argha Manna, Shilpi Saha, Suchismita Mohanty, Shravanti Mukherjee, Minakshi Mazumdar, Deblina Guha, Tanya Das

**Affiliations:** Division of Molecular Medicine, Bose Institute, P-1/12, Calcutta Improvement Trust Scheme VII M, Kolkata, 700054 West Bengal India

**Keywords:** Aspirin, E-cadherin, Migration, NSCLCs, Oncogenic K-ras, p65NFκB

## Abstract

**Background:**

Cancer metastasis is one of the most common causes of treatment failure and death in cancer patients. It has been acknowledged that aberrant activation of epithelial-to-mesenchymal transition (EMT) program, endows cancer cells with metastatic competence for which E-cadherin switch is a well-established hallmark. Suppression of E-cadherin by its transcriptional repressor Slug is thus a determining factor for EMT. Here, we aimed at discerning (i) the molecular mechanisms that regulate Slug/E-cadherin axis in oncogenic K-ras-expressing non-small cell lung carcinoma (NSCLC) cells, and (ii) the effect of aspirin in modulating the same.

**Methods:**

The migratory behaviour of NSCLC cell line A549 were deciphered by wound healing assay. Further assessment of the molecular mechanisms was done by western blotting, RT-PCR, confocal microscopy, chromatin immunoprecipitation and small interfering RNA (siRNA)-mediated gene silencing.

**Results:**

Here we report that in oncogenic K-ras-expressing A549 cells, Ras/ERK downstream Elk-1 forms p-Elk-1-p300 complex that being directly recruited to *SLUG* promoter acetylates the same to ensure p65NFκB binding for transcriptional up-regulation of Slug, a transcriptional repressor of E-cadherin. Aspirin inhibits EMT and decelerates the migratory potential of A549 cells by down-regulating Slug and thereby up-regulating E-cadherin. Aspirin impedes activation and nuclear translocation of p65NFκB, essential for this transcription factor being available for *SLUG* promoter binding. As a consequence, Slug transcription is down-regulated relieving A549 cells from Slug-mediated repression of E-cadherin transcription, thereby diminishing the metastatic potential of these oncogenic Ras-expressing NSCLC cells.

**Conclusions:**

Cumulatively, these results signify a crucial role of the anti-inflammatory agent aspirin as a novel negative regulator of epithelial-to-mesenchymal transition thereby suggesting its candidature as a promising tool for deterring metastasis of highly invasive K-ras-expressing NSCLC cells.

**Electronic supplementary material:**

The online version of this article (doi:10.1186/s12885-016-2078-7) contains supplementary material, which is available to authorized users.

## Background

The major cause of severity and incurability of NSCLCs is due to establishment of cancer cells as metastatic entity. The initiation of tumor metastasis involves increased migratory and invasive capabilities. During tumor progression, tumor cells acquire molecular expression of mesenchymal markers and loss the expression of epithelial markers to result in epithelial to mesenchymal transition (EMT) and subsequent tumor metastasis [1]^1^. EMT is a precisely regulated process through which epithelial cells lose polarity and cell to cell junction and gain a fibroblast-like morphology [2]^2^. An important hallmark of EMT, during which the cell skeleton undergoes rearrangement, is the loss of expre**s**sion of the cell to cell adhesion molecule, E-cadherin which is one of the most important indicators of epithelial phenotype [3]^3^. E-cadherin is thus a suppressor of invasion and metastasis and its down-regulation provokes the development of malignant epithelial cancers [4–6]^4^,^5^,^6^. Several developmentally important genes that induce EMT have been shown to act as E-cadherin repressors [7]^7^. Suppression of E-cadherin expression by its transcriptional suppressor—Slug plays a crucial role in epithelial to mesenchymal transition. Slug, a member of the Snail family of transcriptional repressors, is capable of repressing E-cadherin expression in epithelial cells via the E-box elements in the proximal E-cadherin promoter [7]^8^ and thereby triggering EMT [7–9]^9^,^10^,^11^, suggesting that it may act to promote invasion. Slug expression has been shown to have a strong correlation with loss of E-cadherin in cancer cells [7]^12^, suggesting Slug to be a likely in vivo repressor of E-cadherin expression in metastatic cancers.

It is well known that, human cancers with oncogenic mutation in *Ras* allele are highly aggressive and are associated with poor prognosis. K-ras mutational status has been found to be closely associated with both primary tumors and metastases for more than 90 % of the patients with lung cancer [10, 11]^13^,^14^. Most K-ras mutations in NSCLCs have been found at codon 12 resulting in constitutive activation of Ras proteins that regulates cell junctions in lung epithelial cells through Cox-2 induction and indulges the process of tumor metastasis [12–14]^15^,^16^,^17^. There are several reports signifying NFκB as an important downstream target of Ras-activated signal transduction pathways [15]^18^. Interestingly, correlation between increased activity of NFκB and expression of K-ras has been revealed in recent years [16, 17]^19^,^20^. In fact the activity of transcriptional activation domain of NFκB, i.e., RelA/p65 subunit, was found to be increased significantly in Ras-transformed cells [18]^21^. In an oncogenic K-ras-induced lung cancer mouse model, genetic alteration of p65 has been found to reduce tumorigenesis [19]^22^. Arsura et al. has reported aberrant activation of classical NFκB in Ras-transformed rat liver epithelial cells due to increased phosphorylation and degradation of IκBα protein [20]^23^. Many reports also indicate the involvement of RelA/p65 in metastatic potential of tumors [21–23]^24^,^25^,^26^. According to Huber et al., while NFκB plays a crucial role in the induction of EMT in Ras-transformed mammary epithelial cells, blocking NFκB activity suppresses EMT phenotype [24]^27^. However, the exact molecular mechanism underlying the contribution of p65NFκB in oncogenic K-ras-expressing NSCLC cells invasive responses like EMT and metastasis, for which E-cadherin is a key inhibitory factor, is yet to be delineated.

Accumulating clinical and epidemiological evidences also provides a quite clear and strong link between inflammation and cancer progression. The non-steroidal anti-inflammatory drug aspirin is recently being reported to reduce risk of cancer initiation and progression and suggested to be used to target several tumor properties, including tumor cell migration [25]^28^. Regular use of aspirin has also been observed to decrease the risk of non-small cell lung carcinoma [26–28]^29^,^30^,^31^, thereby suggesting that NSCLCs could be targeted by using aspirin. However, there is no detailed study on the anti-migratory role of aspirin in EMT and NSCLC cells' migration.

In a recent study, using paired colon cancer cell lines that differ in the expression of mutant K-ras, Wang et al. [29]^32^ identified that Slug is selectively required for the survival of cancer cells with mutant K-ras. They further showed that Slug is regulated by the Ras pathway and is very important for activated Ras induced EMT. This and other findings support Slug as a target for treatment of a broad spectrum of human cancers that have undergone EMT, associated at least in part with mutational activation of Ras [30]^33^.

This study elaborates that Ras-down-stream Elk-1-p300 complex acetylates and unwinds *SLUG* promoter to make it accessible for p65NFκB binding which is a pre-requisite for Slug transcription that subsequently leads to E-cadherin down-regulation. Further exploration focuses on the role of anti-inflammatory agent aspirin in up-regulating E-cadherin to inhibit EMT in oncogenic K-ras-expressing NSCLC cells, A549. In gist, aspirin represses the expression of Slug, a known negative regulator of E-cadherin, by blocking the activation of p65 subunit of NFκB and its translocation to nucleus. As a result, E-cadherin gets up-regulated which in turn decelerates the metastatic potential of these highly metastatic NSCLC cells. We therefore, project FDA-approved anti-inflammatory drug aspirin as a novel negative regulator of epithelial-to-mesenchymal transition, thereby signifying its candidature for deterring cancer metastasis.

## Results

### Oncogenic K-ras induces a pro-invasive EMT phenotype in NSCLC cells in a Slug-dependent pathway involving E-cadherin suppression

**Fig. 1:**
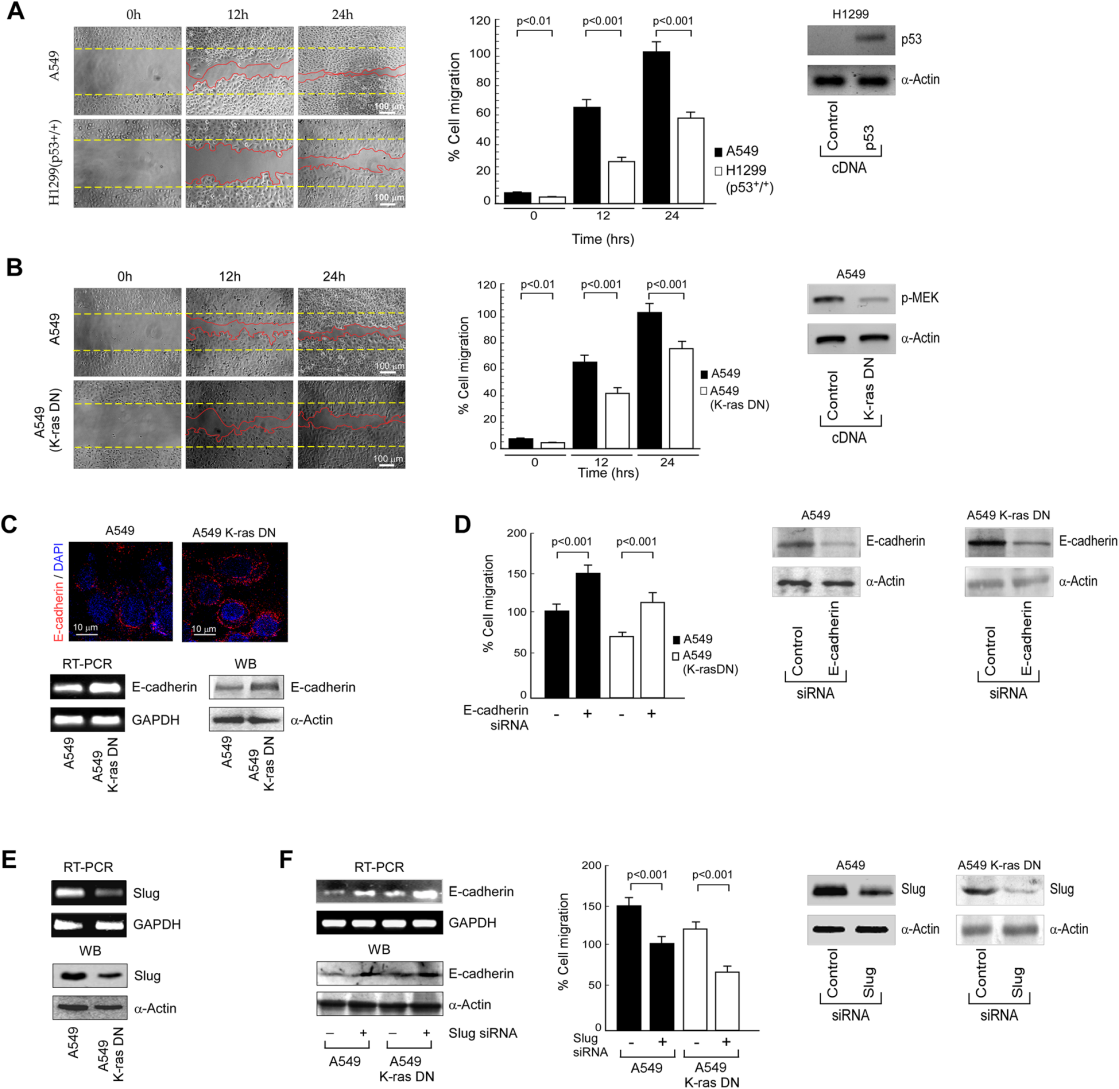
E-cadherin-Slug axis regulates NSCLC cells migration in oncogenic K-ras expressing system. **a** Phase contrast images depicting migration of A549 cells (upper left panel) and p53-reconstituted H1299 cells (lower left panel) for up to 24 h was determined by wound healing assay and percent cell migration was represented graphically (right panel). Scale bar: 100 μm. The inset shows immunoblot analysis for the transfection efficiency of p53-cDNA in H1299 cells. **b** Phase contrast images depict time-dependent migration of oncogenic K-ras expressing control (upper left panel) and DN-K-ras-transfected A549 cells (lower left panel) as determined by wound healing assay and percent cell migration was quantified and represented graphically (right panel). Scale bar: 100 μm. The immunoblot analysis of p-MEK depicts the transfection efficiency of DN-K-ras-cDNA in A549 cells (inset). **c** Upper panel: Expression of E-cadherin was visualised by immunofluorescence in control and DN-K-ras-transfected A549 cells (Scale bar: 10 μm). Images were counterstained with DAPI to represent nuclei. Lower panel: mRNA (left panel) and protein (right panel) expression levels of E-cadherin in control and DN-K-ras-transfected A549 cells as determined by RT-PCR and western blot analyses, respectively. **d** Percent cell migration of untransfected-/ E-cadherin-siRNA transfected, oncogenic K-ras-expressing control and DN-K-ras-reconstituted-A549 cells as determined by wound healing assay was quantified and represented graphically. The inset represents the immunoblot analysis for the transfection efficiency of E-cadherin-siRNA. **e** Expression profiles of Slug mRNA (upper panel) and protein (lower panel) levels of control and DN-K-ras-transfected A549 cells as determined by RT-PCR and western blot analyses, respectively. **f** mRNA (upper left panel) and protein (lower left panel) expression levels of E-cadherin in untransfected-/ Slug-siRNA transfected control and DN-K-ras-reconstituted A549 cells as determined by RT-PCR and western blot analyses, respectively. Graphical representations of percent cell migration for the same experimental set as determined by wound healing assay (right panel). The inset represents the immunoblot analysis for the transfection efficiency of Slug-siRNA. α-Actin/GAPDH was used as an internal loading control. Data are presented as mean ± SEM or representative of three independent experiments

We first sought to evaluate the effects of oncogenic K-ras on the migratory response of wild-type p53-expressing NSCLC cells. To this end, we used A549 cells having K-ras mutation at codon 12 which is responsible for its oncogenic property, and compared its migratory potential with wild type K-ras-bearing H1299 cells that were p53-reconstituted (p53^+^/^+^). Results of wound healing assay showed higher migratory potential of A549 cells than p53-reconstituted H1299 cells (Fig. 1a) suggesting that the metastasizing capacity of cancer cells might be dependent on K-ras status, since all other conditions were same in the two cell lines. Validating this hypothesis, when dominant negative (DN) mutant form of *RAS* (N^17^-*NRAS*) was introduced in A549 cells (Fig. 1b, right panel), reduction in the migratory capacity of these transfectants was observed as compared to parental cells (Fig. 1b, left and middle panels). These results together confirm the contribution of oncogenic K-ras in migration of NSCLC cells. In addition to oncogenic K-ras, mutated p53 are also reported to correlate with metastatic phenotype and play gain-of-function role in oncogenesis. In line with this, two p53 mutants that are commonly found in human cancer and that have been extensively used to study role of p53, in cell migration are R273H (R270H in mice), which directly compromises DNA binding, and R175H (R172H in mice), which causes a global conformational distortion of p53 [31]^34^. These mutations inhibit p53’s ability to act as a transcription factor, accounting for their reduced ability to function as tumor suppressors. For this, to further check the role of mutant p53 in migration of NSCLCs, oncogenic K-ras expressing mutant p53 bearing NSCLC cell line NCI-H522 was used for wound healing migration assay (Additional file 1: Figure S1). Along with this, H1299 cells were transfected with p53 R175H clone (wild type K-ras mutated p53 system) and performed for wound healing assay. Conjointly our data revealed that presence of both oncogenic K-ras and p53 shows highest migratory capacity followed by oncogenic K-ras expressing NSCLCs. Migration potential of mutant p53 follows migration potential of oncogenic K-ras expressing NSCLCs. However, the lowest migration potential among the four sets is showed by wild type p53 expressing wild type Ras bearing sample. This in turn indicates that oncogenic K-ras accelerates the capacity of mutant p53 to induce migration.

Next, we attempted to investigate the mechanism underneath oncogenic K-ras-induced NSCLC cells migration. For the same, we compared the expression of the well known epithelial marker E-cadherin in oncogenic K-ras-bearing parental A549 cells and DN-K-ras reconstituted A549 cells, since loss of E-cadherin has been reported to endorse tumor metastasis [32]^35^. Convincingly, confocal images revealed increase in E-cadherin expression in DN-K-ras reconstituted A549 cells as compared to the parental oncogenic K-ras-expressing A549 cells (Fig. 1c, upper panel). In line with this, RT-PCR (Fig. 1c, lower left panel) and western blot (Fig. 1c, lower right panel) experiments further showed that in contrast to A549 cells, E-cadherin expression increased in DN-K-ras-reconstituted A549 cells. Role of E-cadherin in K-ras mediated retardation of NSCLCs migration was re-confirmed when E-cadherin-siRNA transfected-A549 cells exhibited increased migration over control siRNA-transfected cells (Fig. 1d).

Next, we aimed at exploring the molecular mechanism underneath oncogenic K-ras-mediated inhibition of E-cadherin. Multiple evidences indicate that the metastatic spread of cancer cells is strongly regulated by Slug, a transcriptional repressor of E-cadherin [32]^36^. To evaluate if Slug-dependent E-cadherin repression is relevant for oncogenic K-ras-induced invasiveness, we correlated the levels of Slug expression to K-ras status in parental as well as DN-K-ras-transfected A549 cells. Results of Fig. 1e depicted reduced expression of Slug both at transcriptional (Fig. 1e, upper panel) and translational (Fig. 1e, lower panel) levels in A549 cells with DN-K-ras when compared to parental A549 cells, while silencing Slug (Fig. 1f, right panel) restored E-cadherin levels at both mRNA (Fig. 1f, upper left panel) and protein levels (Fig. 1f, lower left panel), thereby further validating our hypothesis. In a parallel experiment, transfection of DN-K-ras in Slug-silenced (Fig. 1f, right panel) set reduced migration than the control ones (Fig. 1f, middle panel), thereby indicating that oncogenic K-ras uses Slug to induce malignant cell responses.

### Oncogenic K-ras induced migration occurs through transcriptional up-regulation of Slug

**Fig. 2:**
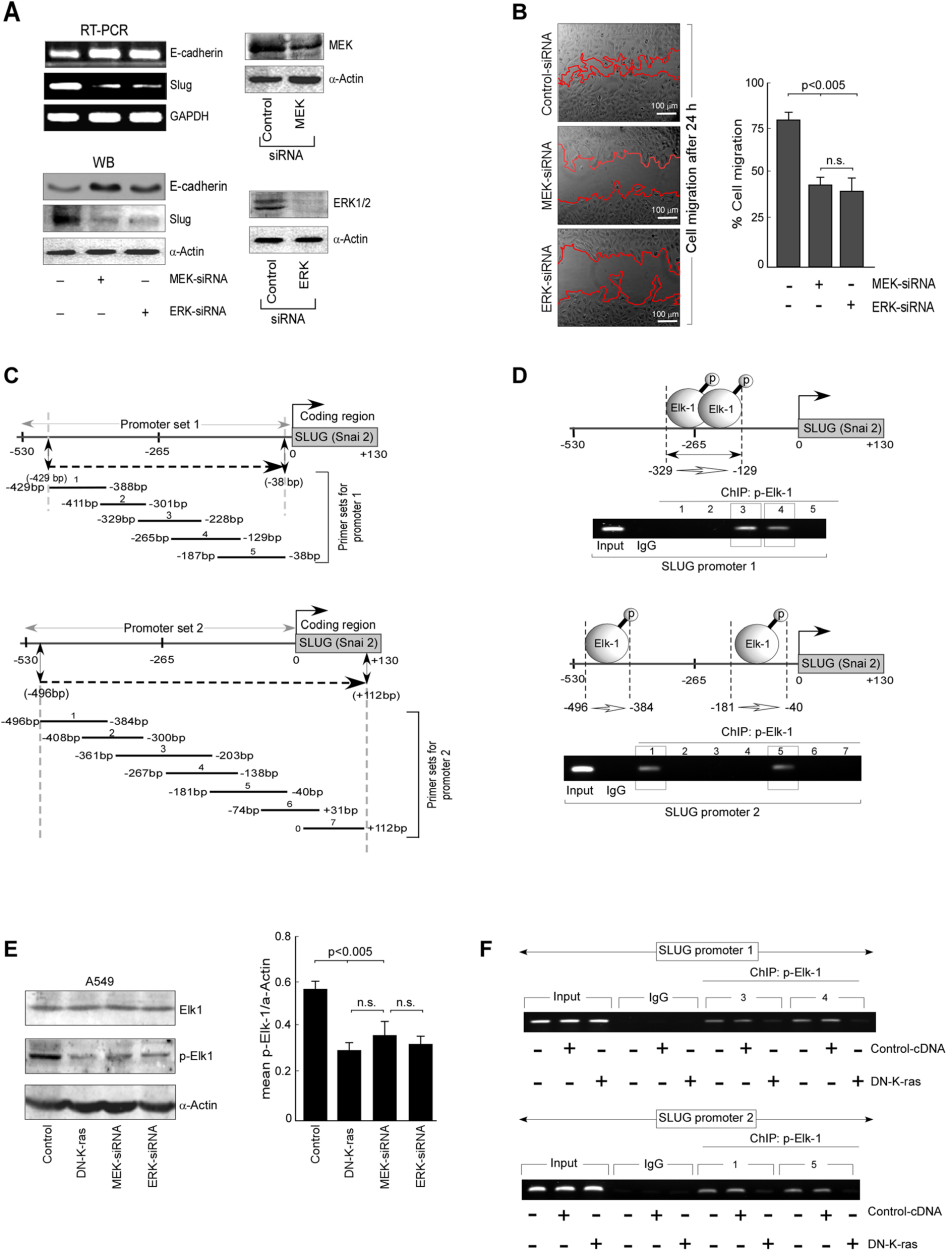
Oncogenic K-ras up-regulates Slug transcriptionally to induce migration in A549 cells. **a** mRNA (upper panel) and protein (lower panel) expression levels of E-cadherin and Slug in untransfected-/ MEK-siRNA-/ ERK-siRNA-transfected A549 cells as determined by RT-PCR and western blot analyses, respectively. The inset represents the immunoblot analysis for the transfection efficiency of MEK-siRNA and ERK-siRNA in A549 cells. **b** Phase contrast images depicting migration of untransfected (upper left panel), MEK-siRNA-(middle left panel) and ERK-siRNA-(lower left panel) transfected A549 cells at 24 h as determined by wound healing assay was quantified and represented graphically (right panel). **c** Schematic diagram representing different regions of *SLUG* promoter and Slug coding region, and the sequential order of primer sets (sets 1–5 for promoter set 1 in upper panel and primer sets 1–7 for promoter set 2 in lower panel) to identify p-Elk-1 binding regions on *SLUG* promoter by ChIP analysis. **d** Schematic representations and RT-PCR data showing p-Elk-1-occupied region on *SLUG* promoter 1 (−325 to −129; primer sets 3 and 4) and *SLUG* promoter 2 (−496 to −384 and −181 to −40; primer sets 1 and 5, respectively) in A549 cells as determined by ChIP analysis. **e** Graphical representation of quantified relative protein expression of p-Elk-1 in untransfected, DN-K-ras-cDNA/MEK-siRNA/ERK-siRNA transfected A549 cells (right pannel) as determined by western blot analysis of Elk-1 and p-Elk-1 (left pannel) (**f**) Chromatin from oncogenic K-ras expressing control A549 cells, control-cDNA-/ DN-K-ras-transfected A549 cells was immunoprecipitated with p-Elk-1 antibody. PCR amplification was performed for p-Elk-1 binding regions on the *SLUG* promoter 1 (upper panel) and *SLUG* promoter 2 (lower panel). α-Actin/ GAPDH served as loading controls. Data are presented as mean ± SEM or representative of three independent experiments

From previous results we hypothesized that, oncogenic K-ras induces migration through up-regulation of Slug. To further investigate the complete signalling pathway up-stream of Slug, we studied downstream components of Ras/Raf/MEK/ERK pathway, MEK and ERK, and silenced them individually with MEK-siRNA (Fig. 2a, upper right panel) and ERK-siRNA (Fig. 2a, lower right panel), respectively, in oncogenic K-ras-bearing A549 cells. Both the transfectants showed increased expression of E-cadherin as well as decreased expression of Slug than control A549 cells, at both mRNA (Fig. 2a, upper left panel) and protein (Fig. 2a, lower left panel) levels. In line with this, percent cell migration was lower for MEK-siRNA- or ERK-siRNA-transfected A549 cells than control ones (Fig. 2b).

Interestingly, although silencing MEK/ERK led to Slug down-regulation, neither MEK nor ERK has any DNA-binding domains and, therefore, is not considered as transcription factors [33]^37^. Considering this information, we next searched for the contribution of another downstream transcription factor and observed the contribution of Elk-1, a direct substrate of ERK [34]^38^, in Slug repression. Moreover, the ETS family transcription factor Elk-1 is known as the downstream effecter molecule of all three MAPK pathways, i.e. p38 ^MAPK^, JNK and ERK pathways but through different residues [35]^39^. ERK can bind to Elk-1 in the D-domain, which is located N-terminal from the C-terminal transcriptional domain (C-domain), and phosphorylate S383 and S389 in this domain [36]^40^. ERK-induced Elk-1 phosphorylation leads to enhanced DNA-binding and TCF-mediated transcriptional activation [37]^41^. Therefore, in our system, we designed battery of overlapping primer sets for *SLUG* promoter (Fig. 2c) and our chromatin immunoprecipitation (ChIP) assay demonstrated four putative binding sites of Elk-1 on the *SLUG* promoter, two on promoter 1 (−329 to −228 and −265 to −129) (Fig. 2d, upper panel) and two on promoter 2 (−496 to −384 and −181 to −40) (Fig. 2d, lower panel). Further search showed that in comparison to the control cells, Elk-1 expression did not show any significant change while the activation of Elk-1 was decreased in both DN-K-ras-expressing, MEK-siRNA-transfected and ERK-siRNA-transfected A549 cells (Fig. 2e, left panel). Since the changes in phophorylation of Elk-1 are relatively small, densitometric analysis of the same was represented in the right panel of Fig. 2e. In our next experiment, binding of p-Elk-1 on *SLUG* promoter was found to be decreased in DN-K-ras-reconstituted A549 cells than oncogenic K-ras-expressing control A549 cells (Fig. 2f). In keeping with our previous experiment where DN-K-ras-reconstituted A549 cells showed down-regulation of Slug mRNA levels than the control ones (Fig. 1e), these results directly proved the role of K-ras-downstream p-Elk-1 for Slug up-regulation.

### Oncogenic K-ras in association with p65NFκB, up-regulates Slug transcription in A549 cells

**Fig. 3:**
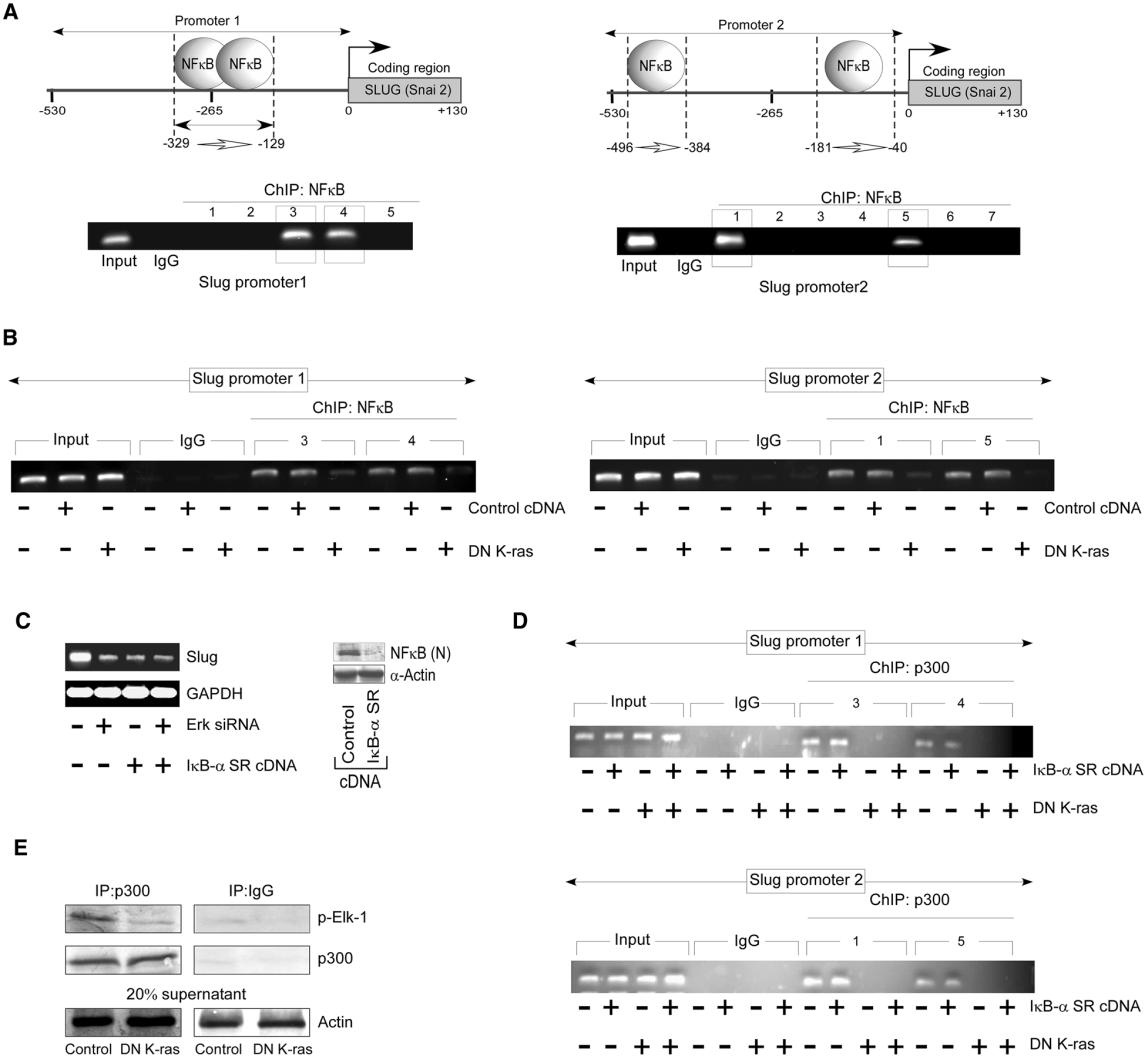
Slug up-regulation in A549 cells occur conjointly with oncogenic K-ras and p65NFκB pathways. **a** Schematic representations (upper panels) and RT-PCR data (lower panel) of p65NFκB-occupied region on *SLUG* promoter 1 (−329 to −129; primer sets 3 and 4) and *SLUG* promoter 2 (−496 to −384 and −181 to −40; primer sets 1 and 5, respectively) in A549 cells as determined by ChIP analysis. Lane 1 denotes input control and parallel immunoprecipitation with control IgG antibody is shown in lane 2. **b** Chromatin from oncogenic K-ras expressing control A549 cells, control-cDNA-/ DN-K-ras-transfected A549 cells was immunoprecipitated with p65NFκB antibody. PCR amplification was performed on p65NFκB binding regions on the *SLUG* promoter 1 (upper left panel) and *SLUG* promoter 2 (upper right panel). Lane 1, 2 and 3 denotes input control and parallel immunoprecipitation with control IgG antibody is shown in lane 4, 5 and 6. (**c**) RT-PCR analysis for Slug mRNA levels in untransfected, ERK-siRNA-/ IκBα-SR-cDNA-transfected A549 cells and in A549 cells transfected with both ERK-siRNA and IκBα-SR-cDNA. The inset shows the immunoblot analysis of nuclear p65NFκB levels for the transfection efficiency of IκBα-SR-cDNA. **d** ChIP assay for p300 binding on the *SLUG* promoters 1 and 2 in untransfected, DN-K-ras-/IκBα-SR-cDNA transfected A549 cells and in A549 cells transfected with both DN-K-ras and IκBα-SR-cDNA. **e** Nuclear lysates of oncogenic K-ras expressing A549 cells and DN-K-ras-transfected A549 cells was subjected to immunoprecipitation using anti-p300 antibody or with control IgG. The same blot was sequentially probed with p-Elk-1 and p300-specific antibodies. To confirm comparable protein input of the respective samples following, 20 % of lysates used for immunoprecipitation were subjected to western blot analysis with anti-α-Actin. Data are presented as mean ± SEM or representative of three independent experiments

It has been documented that in A549 cells, p65NFκB is over-expressed [38]^42^ and p65/RelA subunit of NFκB is functionally activated by Ras for efficiently promoting tumorigenesis [39]^43^. A very recent report also indicates binding of p65NFκB on *SLUG* promoter in A549 cells [40]^44^. Keeping these in mind, we next aimed at determining whether the interaction between Elk-1 and p65NFκB influences Slug expression and the mechanism behind it. Our search revealed four putative p65NFκB binding sites adjacent to p-Elk binding sites on *SLUG* promoter (Fig. 3a). We further aimed at verifying whether absence of oncogenic K-ras hampers binding of p65NFκB to *SLUG* promoter or not. Our ChIP analysis showed inhibition in p65NFκB binding in DN-K-ras reconstituted set (Fig. 3b). Next to check whether bindings of both oncogenic K-ras and p65NFκB are essential for Slug transcription to occur, we inhibited p65NFκB nuclear translocation by transfecting A549 cells with IκBα-SR-cDNA or silenced Elk-1-upstream ERK by transfecting A549 cells with ERK-siRNA (Fig. 3c). In both the cases, Slug transcription was found to be down-regulated similarly than control A549 cells (Fig. 3c). These results depict that the presence of both active p65NFκB and p-Elk-1 is pre-requisite for Slug expression to occur.

To understand the exact molecular mechanism underlying chromatin modification in our system, we checked the acetylation status of *SLUG* promoter under different conditions. To that end, either DN-K-ras or IκBα-SR-cDNA was transfected individually or in combination. In the control cells, where both oncogenic K-ras and p65NFκB were present, and in IκBα-SR-cDNA-transfected sets acetylation of *SLUG* promoter was observed. However, in cells transfected with DN-K-ras alone or in combination with IκBα-SR-cDNA, no acetylation of *SLUG* promoter could be seen (Fig. 3d). Since, in DN-K-ras transfected set, chromatin acetylation was absent and p65NFκB binding was hampered, these results highlighted that p-Elk1 is responsible for chromatin acetylation and thus for p65NFκB binding on *SLUG* promoter.

Since, Elk-1 is reported to form a pre-assembled complex with p300 and p300 acts like a co-activator in complexes that contain Elk-1, we proposed that in our system Elk-1 recruits p300 to acetylate *SLUG* promoter. Our co-immunoprecipitation experiment with p-Elk-1 and p300 confirmed our hypothesis (Fig. 3e). All these results further validated the co-operation of p-Elk-1 and p65NFκB for Slug up-regulation. Moreover, the parallel between gain of anti-migratory functions of oncogenic K-ras and p65NFκB is compatible with the concept of mutant K-ras acting as a promoting factor for the tumor migratory activity of p65NFκB.

### Aspirin inhibits migration through down-regulation of Slug and up-regulation of E-cadherin

We next aimed to use FDA approved non-steroidal anti-inflammatory drug aspirin, which is a known p65NFκB inhibitor [41, 42]^45^,^46^ and is reported to reduce risk for cancer initiation and progression to its users [43–45]^47^,^48^,^49^. Additionally, several reports suggest that aspirin could be targeted for several other tumor properties including tumor migration [46–48]^50^,^51^,^52^. However, there are no detail studies indicating anti-migratory role of aspirin on NSCLCs.

**Fig. 4:**
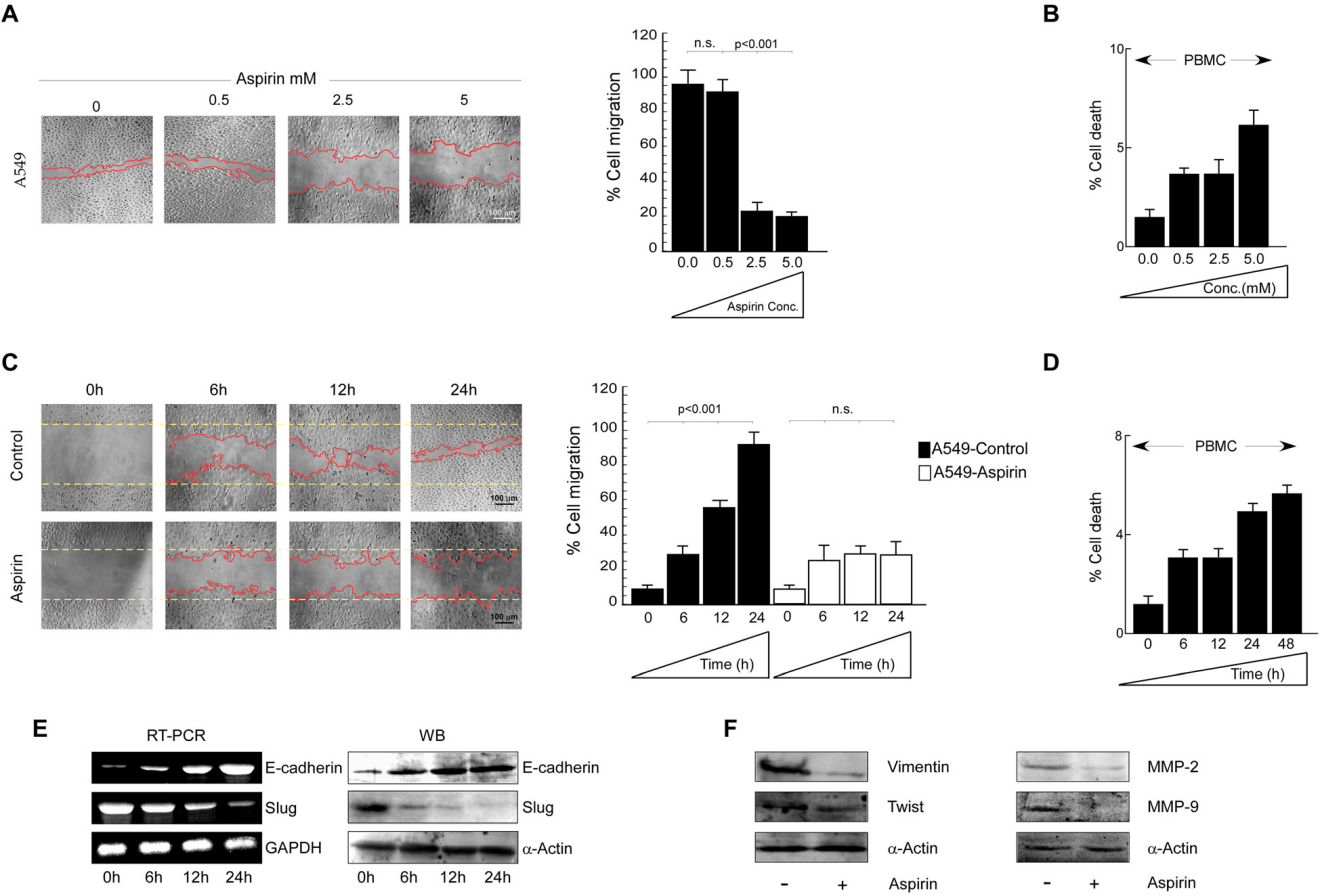
Aspirin retards migration of A549 cells by restoration of E-cadherin expression. **a** Phase contrast images depicts migration of A549 cells upon 24 h treatment with aspirin in a dose-dependent manner (left panel) was determined by wound healing assay and percent cell migration was represented graphically (right panel). Scale bar: 100 μm. **b** Graphical representation of percent cell death in PBMCs upon treatment with aspirin in a dose-dependent manner. **c** Phase contrast images depicting migration of untreated (upper left panel) and aspirin (2.5 mM)-treated (lower left panel) A549 cells in a time-dependent manner was determined by wound healing assay and percent cell migration was represented graphically (right panel). Scale bar: 100 μm. **d** Graphical representation of percent cell death in PBMCs upon treatment with 2.5 mM aspirin in a time-dependent manner. **e** Expression profiles of E-cadherin and Slug mRNA (left panel) and protein (right panel) levels of A549 cells upon treatment with aspirin in a time dependent manner as determined by RT-PCR and western blot analysis, respectively. **f** Protein expression profiles of migration related markers, Vimentin, Twist, MMP-2 and MMP-9 in untreated and aspirin-treated A549 cells as determined by western blot analysis. α-Actin/ GAPDH served as loading controls. Data are presented as mean ± SEM or representative of three independent experiments

Interestingly, results of Fig. 4a depicted decrease in percent cell migration with increasing doses of aspirin (0, 0.5, 2.5 and 5 mM). Since, recent report indicates that aspirin reaches human plasma level at only 2.5 mM concentration [49]^53^; we used same concentration for our further experiments. Importantly, this dose was observed to be non-toxic towards normal cell like PBMCs (Fig. 4b), thereby verifying this as the effective non-toxic anti-migratory dose of aspirin. Results of Fig. 4c further showed the time-dependence of the anti-migratory effect of aspirin 2.5 mM concentration. Interestingly, 2.5 mM of aspirin was also found to be non-toxic for upto 24 h (Fig. 4d).

Next, to explore whether this aspirin-induced anti-migratory effect is mediated by Slug/E-cadherin axis, we performed RT-PCR and western blotting for E-cadherin and Slug with or without 2.5 mM dose of aspirin (Fig. 4e). Interestingly, while expressions of both E-cadherin mRNA (Fig. 4e, left panel) and protein (Fig. 4e, right panel) were induced upon aspirin treatment, those of Slug were decreased in aspirin-treated sets (Fig. 4e). In addition, expressions of other EMT marker proteins, vimentin, twist, MMP-2 and MMP-9, were down-regulated by aspirin (Fig. 4f). These results together indicated that the non-toxic anti-migratory dose of aspirin induced epithelial markers and consequently repressed mesenchymal markers to restrain migration of A549 cells.

### Aspirin ensures Slug down regulation by inhibiting p65NFκB - nuclear translocation

**Fig. 5:**
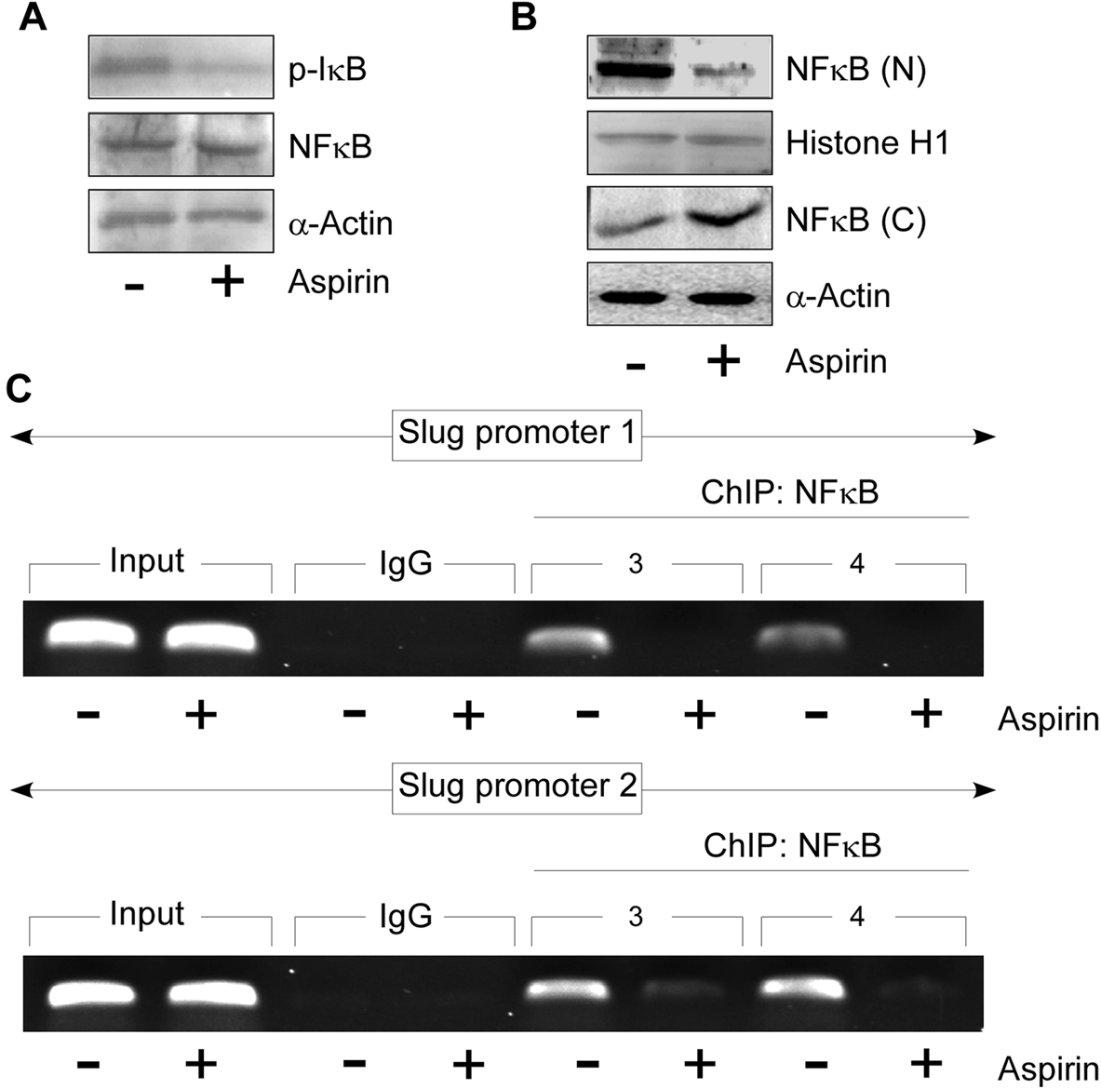
Aspirin retards p65NFκB-nuclear translocation to ensure Slug down-regulation. **a** Western blot analysis to determine p-IκBα and total p65NFκB expression levels in untreated and aspirin treated-A549 cells. **b** Protein expression levels of p65NFκB in nuclear and cytosolic lysates of untreated and aspirin treated-A549 cells as determined by western blot assay. **c** ChIP assay for p65NFκB binding on the *SLUG* promoter 1 (upper panel) and 2 (lower panel) in untreated and aspirin treated-A549 cells. α-Actin/ Histone H1 served as loading controls. Data are presented as mean ± SEM or representative of three independent experiments

NFκB plays a central and evolutionarily conserved role in coordinating the expression of various soluble pro-inflammatory mediators and leukocyte adhesion molecules. Several reports suggest that aspirin impedes cancer initiation and progression by inhibiting activation of NFκB pathway [50]^54^. Results of Fig. 5a demonstrated reduction in phosphorylation of IκBα upon aspirin treatment, total p65NFκB expression remaining same. This indulged us to further investigate whether aspirin constrains nuclear translocation of p65NFκB or not. Next in Fig. 5b we observed reduced nuclear expression of p65NFκB with increase in its cytosolic expression in A549 cells upon aspirin treatment (Fig. 5b). These results together highlight that aspirin treatment impedes activation and therefore nuclear translocation of p65NFκB in A549 cells.

Concurrently, ChIP analysis further depicted reduced binding of p65NFκB on *SLUG* promoter in aspirin-treated A549 cells as compared to the untreated ones (Fig. 5c). These results in correlation with our previous data suggest that aspirin retracts p65 NFκB-induced Slug transcription thereby inhibiting NSCLC cells migration.

In summary, Aspirin works to ensure Slug down-regulation by restraining NFκB nuclear translocation, a pre-requisite for *SLUG* promoter activation, in oncogenic K-ras expressing NSCLCs.

## Discussion

The disappearance of epithelial phenotype and acquisition of mesenchymal phenotype constitute the basic molecular and morphological manifestations of EMT. This process increases cell mobility and constitutes a critical step in cell migration, which is associated with various biological processes, including cancer invasion and metastasis. Thus, the maintenance of epithelial phenotype and suppression of EMT have been increasingly recognized to be important for preventing cancer progression. During the execution of the EMT program many genes involved in cell adhesion, migration and invasion are transcriptionally altered, E-cadherin being one of the most important [51]^55^. Since E-cadherin functions as a key gatekeeper of the epithelial state, the partial loss of E-cadherin has been associated with carcinoma progression and poor prognosis in various human and mouse tumors [52]^56^. Evaluation of the molecular mechanisms involved in regulation of E-cadherin expression, therefore, might be a critical step in controlling EMT. Our present study has been mainly focused on the regulation of EMT by altering E-cadherin expression by the NSAID aspirin in oncogenic Ras-expressing NSCLC cells. Aspirin enhanced E-cadherin expression, which was conjointly associated with a loss in the migratory potential of these cells, via down-regulation of E-cadherin repressor Slug by inhibition of p65NFκB activation and its translocation to nucleus for *SLUG* promoter binding.

Our effort to explore the effects of oncogenic K-ras in aggravation of migration of NSCLC cells revealed that oncogenic K-ras-bearing NSCLCs induce migration via Slug/E-cadherin axis. In line with these findings there are reports describing that expression of oncogenic K-ras induces EMT and confers a metastatic phenotype on carcinomas by repressing the E-cadherin gene at the transcriptional level [53]^57^. Consistently, Shin et al. has demonstrated the implication of Ras-ERK signaling in Ras-induced transformation of epithelial cells into mesenchymal cells [54]^58^. Our results also showed that Ras/Raf downstream effector molecule Elk-1 plays a crucial role in NSCLC cells migration. In depth analysis denoted that Elk-1 gets activated by Ras/Raf/ERK pathway and forms a complex with p300 to acetylate *SLUG* promoter, thereby accelerating transcriptional activation of Slug. Supporting our observation, report of Li et al. evidenced that Elk-1 forms pre-assembled Elk-1-p300 complex which become active following phosphorylation of Elk-1 to ultimately lead to target gene transcription [55]^59^. That Elk-1 can interact with p300 both in vitro and in vivo through the C-terminus of Elk-1 and the N-terminus of p300 has already been documented [55]^60^. Such changes in interaction render a strong histone acetyltransferase activity in the Elk-1-associated complex that could play a critical role in chromatin remodelling and gene activation [55]^61^.

In previous studies, it has been revealed that in oncogenic K-ras-induced lung cancer mouse model, genetic alteration of p65 subunit of NFκB reduces tumorigenesis (19)^62^ thereby substantiating the contribution of p65NFκB in activated Ras-induced tumor formation. Indeed, genetic alteration of p65 leads to the tumor regression in K-ras-driven mouse tumor models [19]^63^. In contrast, activity of transcriptional active domain, i.e., p65NFκB, was found to be increased strikingly in Ras/Raf-transformed cells [20]^64^. However, such mutual interaction between the two oncogenic pathways in promoting cancer cell migration is yet to be revealed. Given that oncogenic K-ras mutations co-exist with p65NFκB over expression in A549 cells [38]^65^, we postulated that K-ras/NFκB axis might be an important target to curb migration in these NSCLC cells. Our findings that p65NFκB plays a pivotal role as a transcription factor to up-regulate Slug transcription for migration of oncogenic K-ras-expressing A549 cells indicated the possibility of EMT being regulated by the conjoint effort of both K-ras and p65NFκB pathways in these cells. While K-ras pathway brings about the acetylation of the *SLUG* promoter, commencement of Slug transcription occurs only through the binding of the transcription factor p65NFκB. Reports are there in support of Slug activation by NFκB to confer resistance to TNF-α-induced apoptosis in A549 cells [56]^66^. Further support comes from the role played by IKKα, inhibitor of NFκB that controls canonical TGFβ–SMAD signaling to regulate genes expressing Snail and Slug during EMT in pancreatic cancer cell line [57]^67^. Several other findings together with these suggest Slug as a molecular platform or a target for treatment of a range of metastatic cancers associated with mutational Ras.

The present study further signifies the effect of FDA-approved non-steroidal anti-inflammatory drug aspirin on highly invasive and migratory NSCLC cell line A549. Aspirin, i.e., acetyl salicylic acid, is known for decades to inhibit transcription of several genes including adhesion molecules and nitric oxides, which are known to regulate inflammatory pathways [58]^68^. One of such well studied targets of aspirin is p65NFκB [50]^69^, activation of which is associated with tumor progression and metastasis of several human tumor types [59]^70^. Yet, the effect of aspirin on the activation of p65NFκB differs according to cell type. Present study discusses the anti-migratory effect of this anti-inflammatory drug on lung epithelial cell A549. We observed that aspirin inhibits A549 cell migration by suppressing p65NFκB activation and translocation to the nucleus for binding to *SLUG* promoter, thereby resulting in the transcriptional down-regulation of Slug in these NSCLC cells. Our observation that aspirin restrains constitutively active NFκB in lung cancer cells concurs with recent studies demonstrating that aspirin can also inhibit inducible p65NFκB in cervical cancer and hepatoma cells [60]^71^.

**Fig. 6:**
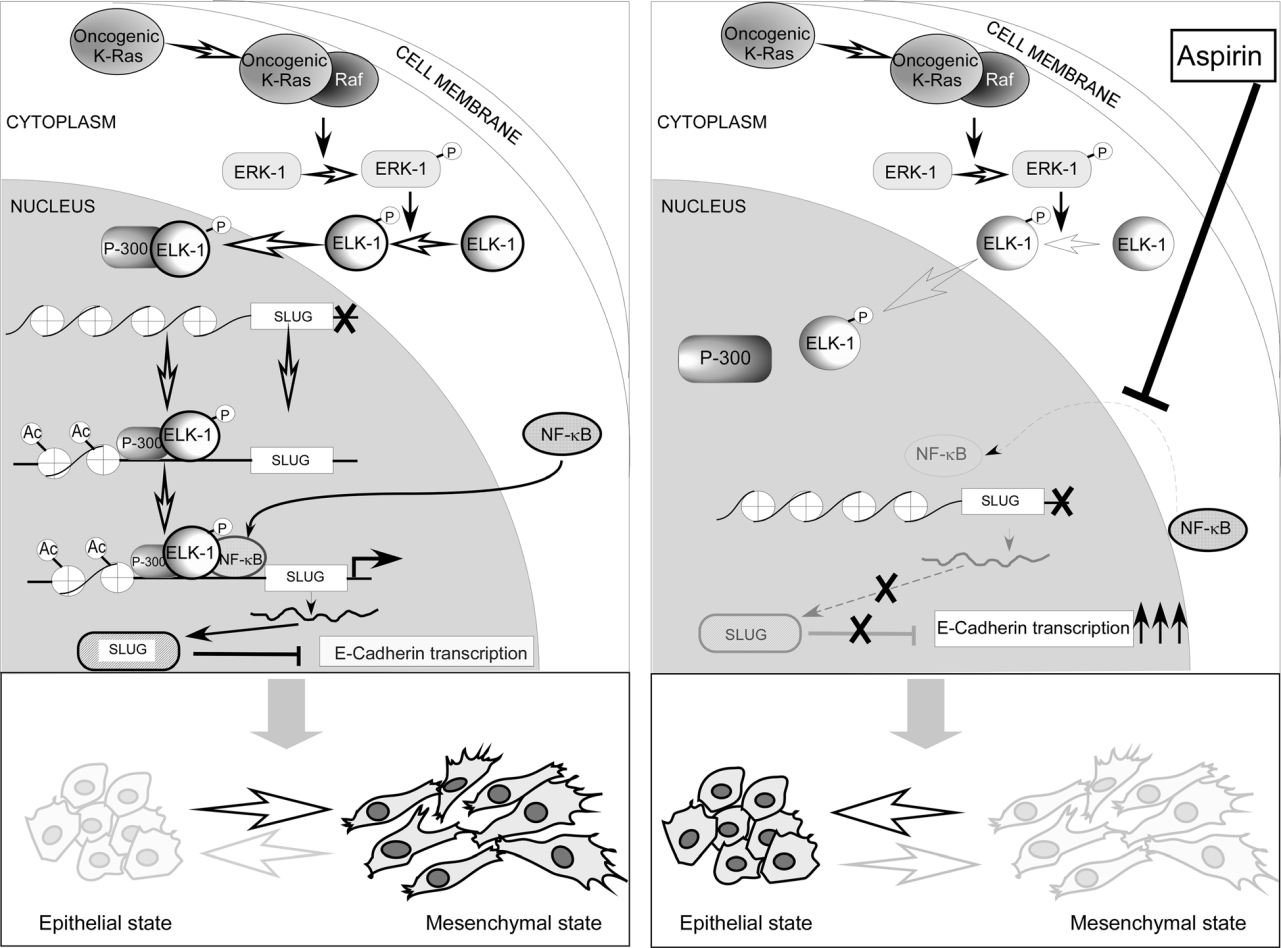
Schematic illustration depicts the potential role of aspirin in reprogramming EMT in oncogenic K-ras-expressing NSCLC cells. Left panel demonstrates the conjoint role of K-ras and NFκB pathways in up-regulation of Slug transcription towards EMT in NSCLC cells. Right panel shows aspirin-mediated inhibition of EMT and migration of NSCLC cells through inhibition of p65NFκB nuclear translocation that resulted in down-regulation of Slug, a transcriptional repressor of E-cadherin

Collectively, this study provides a complex molecular framework involving p65NFκB during MEK/ERK-mediated EMT. In that molecular network, p-Elk-1-p300 complex induces histone acetylation and unwinding of *SLUG* promoter to make access for NFκB on the same. Binding of p65NFκB on *SLUG* promoter, in turn, ensures Slug transcriptional up- regulation and subsequent Slug-dependent E-cadherin repression. In contrast, aspirin impedes nuclear translocation of p65NFκB thereby repressing Slug and consequently restoring E-cadherin levels. Therefore, by modulating the pro-migratory molecular architecture, aspirin nullifies the effect of oncogenic Ras-induced migration of NSCLCs. Altogether this study highlights the complexity of gene-regulation and demonstrates how aspirin abrogates effectors of EMT such as K-ras, which, via epigenetic alterations through a well-known MEK/ERK pathway, adds to p65NFκB functions to cause tumor metastasis (Fig. 6). Such activities of aspirin strongly support its candidature as a potential anti-migratory agent, suggesting the possibility of development of a treatment regimen in future for highly metastatic non-small cell lung carcinoma.

## Conclusion

This preclinical study suggests aspirin as a potent anti-migratory agent to improve the therapeutic index of highly metastatic NSCLCs in which EMT, a pre-requisite for cancer cell migration, being programmed by the conjoint effort of both K-ras and NFκB pathways. Aspirin inhibited EMT and delayed migration of oncogenic K-ras-expressing NSCLC cells through inhibition of p65NFκB nuclear translocation that resulted in down-regulation of Slug, a transcriptional repressor of E-cadherin. Knowledge gathered from this study may open a new avenue in future for developing more effective treatment strategy for controlling highly invasive NSCLCs with oncogenic K-ras.

## Methods

### Cell culture and treatments

The non small cell lung cancer cell line A549 (K-ras v-12 mutated) H1299 and NCI-H522 were obtained from National Centre for Cell Science, Pune, India. The cells were routinely maintained in complete Dulbecco’s modified Eagle’s medium at 37 °C in a humidified incubator containing 5 % CO_2_ [61]^72^. Cells were allowed to reach confluency before use. Viable cell numbers were determined by Trypan blue exclusion test. Since this study involved only human cell lines and no human or animal participants or tissues, ethical clearance was not required.

### Treatment of cells

Exponentially growing human non-small cell lung carcinoma cell line-A549 were seeded at a density of 2.5x10^6^ cells/100 mm culture dishes (Becton Dickinson, Franklin Lakes, NJ) 24 h before aspirin treatment. Cells were treated with different concentrations of aspirin (Acetyl Salicylic Acid from MP Biomedicals) for different time points to select the optimum dose and time required to reduce cancer cell migration. 1 M stock solution of aspirin was prepared in DMSO and stored at −20 °C. Now, to reach final concentrations of 0.5, 2.5 or 5 mM of aspirin, 0.5 μL, 2.5 μL or 5 μL of stock solution was added, respectively, in 1 ml cell culture media. An equivalent amount of carrier (DMSO) was added to untreated cells as control to ensure that the observed effect was related to aspirin and not due to the solvent.

### Wound healing assay

Cell migration was determined by means of bidirectional wound healing assay as described [62]^73^. Briefly, cells were grown to confluency in 12 well plates after which a sterile blade was used to scratch the monolayer of cells to form a bidirectional wound. Migration was quantitated by a semi-automated, computer-assisted procedure by a person blinded with respect to the experimental treatment. The data from triplicate wells were calculated as the mean ± S.E.M., the migration rate of control cells was taken as 100 % and healing rate of other plates were compared with control cells.

### Immunofluorescence

For immunofluorescence, cells were grown on sterile glass coverslips at 37 °C for 24 h. Cells after treatment were washed briefly with PBS and fixed with 4 % formaldehyde for 20 min at 37 °C. Thereafter, cells were blocked for 2 h in a blocking buffer (10 % BSA in PBS) and then additionally incubated for another hour in PBS with 1.5 % BSA containing anti- E-cadherin antibody (Santa Cruz, CA, USA). After washing in PBS, cells were incubated with PE conjugated secondary antibody in PBS with 1.5 % BSA for 45 min at 37 °C in the dark. DAPI was used for nuclear staining. Coverslips were washed with PBS and mounted on microscopy glass slides with 90 % glycerol in PBS. Images were acquired using a confocal microscope (Carl Zeiss, Jena, Germany) [63]^74^.

### Immunoblotting and co-immunoprecipitation

To obtain whole cell lysates, cells were homogenized in lysis buffer (20 mM Hepes, pH 7.5, 10 mM KCl, 1.5 mM MgCl2, 1 mM Na-EDTA, 1 mM Na-EGTA and 1 mM DTT) supplemented with protease and phosphatase inhibitor cocktails [64, 65]^75^,^76^. For direct western blot analysis, a total of 50 μg of protein was resolved using SDS-PAGE and transferred to nitrocellulose membrane and probed with specific antibodies, for example, anti-NFκB/-IκB/-slug/-E-cadherin/-MEK/-ERK/-Elk-1/-p-Elk-1/-p53(DO-1)/-p53(FL-393) antibodies (Santa Cruz, CA, USA), thereafter the immunoblots were visualized by chemiluminescence (GE Biosciences, NJ, USA). To study the interaction between Elk1 and p300, p300 immunocomplex from whole cell lysate was purified using p300 antibody and protein A-Sepharose beads (Invitrogen, MD). The immunopurified protein was immunoblotted with Elk-1 antibody. The protein of interest was visualized by chemiluminescence. Equal protein loading was confirmed with anti-α-actin and histone H1 antibodies (Santa Cruz, CA, USA) [66]^77^.

### RT–PCR assay

Two microgram of the total RNA, extracted from cells with TRIzol Q16 reagent (Invitrogen, Carlsbad) was reverse transcribed and then subjected to PCR with enzymes and reagents of the RTplusPCR system (Eppendorf, Hamburg, Germany) using GeneAmpPCR 2720 (Applied Biosystems, CA, USA). The cDNAs were amplified with primers specific for E-cadherin (5′-GTCATCCAACGGGAATGCA-3′/5′-TGATCGGTTACCGTGATCAAAA-3′), Slug (5′-CTCACCTCGGGAGCATACAG-3′/5′-GACTTACACGCCCCAAGGATG-3′), GAPDH (internal control): (5′-CAGAACATCATCCTGCCTCT-3′/5′-GCTTGACAAAGTG GTCGTTGAG-3′) [67, 68]^78^,^79^.

### Plasmids, siRNA and transfections

The expression constructs p53-cDNA, p53-R175H cDNA (kind gift from Moshe Oren, Weizmann Institute of Science), N^17^-*NRas*, IκB-SR and control pcDNA3.0 vectors (2 μg/million cells) were introduced into exponentially growing cells using lipofectamine-2000 (Invitrogen, Carlsbad) according to the protocol provided by the manufacturer. Stably expressing clones were isolated by limiting dilution and selection with G418 sulfate (1 mg/ml; Cellgro) and G418 resistant cells were cloned and screened by immunoflourescence or western blotting with specific antibodies. For endogenous silencing of specific genes, cells were transfected with 300 pmol of E-cadherin-/Slug-/MEK-/ERK-/control-siRNA using lipofectamine-2000 separately for 12 h. The mRNA and protein levels were determined by RT-PCR and western blotting, respectively [69]^80^.

### Chromatin immunoprecipitation (ChIP)

ChIP assays were performed using a ChIP assay kit (Millipore, Darmstadt, Germany) according to the manufacturer’s instructions. PCR assay for identification of p-Elk-1 and NFκB binding regions on *SLUG* promoter was performed using the 12 different primer sets.

For promoter1:

1. 5′GACCCATACAACCCTTTTTCC3′/5′GGAACCACCGGACATTCTCT3′;
2. 5′GTGAGAGAATGTCCGGTGGT3′/5′CTCTAAAGGCAGGCTGATCG3′;
3. 5′TTCCAGTTCTTCCGATCAGC3′/5′GCCGCGTGCAAATTAAGTA3′;
4. 5′CTAACACGGTGACATGAGTAC3′/5′GACGCTCTCCTGGGACTCTG3′;
5. 5′CTCCAGGCCAGAGTCCCAG3′/5′GTTTGCCTTGCACAAAGACC3′.

For promoter 2:

1. 5′CGCTTCCCCCTTCCTTTTTC3′/5′CAGCCTCTGGTGTTAATGAGAGC3′;
2. 5′GGCTCTCATTAACACCAGAGG3′/5′CTGGCTTCAAGATGTGTTGCAG3′;
3. 5′CTTCCTTCTCCTTGCGAACAC3′/5′CAAGAGAGGTAACATCGCTCGG3′;
4. 5′CCCTCCTAGCTTCCAGAGAG3′/5′TCTGGTTCAAAATGGGCTG3′;
5. 5′CCTCTCCACGGAAATCTCAA3′/5′GCAAGAAAGATCCAAGCACAGC3′;
6. 5′CTGAACCTCTCAACTGTGATTGG3′/5′CTCTGAAGTCAACCGGCTC3′;
7. 5′CAGTTCGTAAAGGAGCCGG3′/5′CACGGCGGTCCTTAAAGCATC3′.

Extracted DNA (2 μl) was used for 45 cycles of amplification in 50 μl of reaction mixture under the following conditions: 95 °C for 30s, 56 °C for 30s, and 72 °C for 60 s. The PCR products were analysed by 2 % agarose gel electrophoresis [70]^81^.

### Statistical analysis

Values are shown as standard error of mean, except otherwise indicated. Data were analyzed and, appropriate, significance (*p* < 0.05) of the differences between mean values was determined by a Student’s *t* test.

## Additional file

Additional file 1: Figure S1. p53 mutation exerts increased migratory effect in combination with oncogenic K-ras-expressing system on NSCLC cells migration. Phase contrast images (left panels) depicting migration of NCI-H522 cells (oncogenic K-ras/mutant p53), mutant p53-reconstituted H1299 cells (wild type K-ras/mutated p53), A549 cells (oncogenic K-ras/wild type p53), and wild type p53 reconstituted H1299 cells (wild type K-ras/wild type p53). Graphical representation of percent cell migration in wound healing assay (right panel) with inset showing immunoblot analysis for the transfection efficiency of p53 - R175H clone in H1299 cells and p53 - cDNA in H1299 cells. Scale bar: 100 μm. (TIFF 2457 kb)

## Abbreviations

DN-K-ras: Dominant negative K-ras
EMT: Epithelial-mesenchymal transition
IκBα-SR cDNA: Inhibitory kappa beta super repressor complementary DNA
MMP-2: Matrix metalloproteinase-2
MMP-9: Matrix metalloproteinase-9
NSAID: Non-steroidal anti-inflammatory drug
NSCLC: Non-small cell lung carcinoma
PE: Phycoerythrin

## Footnotes

1 Thiery JP. Epithelial-mesenchymal transitions in tumour progression. Nat Rev Cancer. 2002;2(6):442–454.

2 Lamouille S, Xu J, Derynck R. Molecular mechanisms of epithelial–mesenchymal transition. Nat Rev Mol Cell Biol. 2014;15(3):178–196.

3 Singhai R, Patil VW, Jaiswal SR, Patil SD, Tayade MB, Patil AV. E-Cadherin as a diagnostic biomarker in breast cancer. N Am J Med Sci. 2011;3(5):227–233.

4 Mareel M, Leroy A. Clinical, cellular and molecular aspects of cancer invasion. Physiol Rev. 2003;83(2):337–376.

5 Bremnes RM, Veve R, Gabrielson E, Hirsch FR, Baron A, Bemis L, et al. High-throughput microarray analysis used to evaluate biology and prognostic significance of the E-cadherin pathway in small cell lung cancer. J Clin Oncol. 2002;20(10):2417–2428.

6 Frixen UH, Behrens J, Sachs M, Eberle G, Voss B, Warda A, et al. E-cadherin-mediated cell-cell adhesion prevents invasiveness of human carcinoma cells. J Cell Biol. 1991;113(1):173–185.

7 Hajra KM, Chen DY, Fearon ER. The SLUG zinc-finger protein represses E-cadherin in breast cancer. Cancer Res. 2002;62(6):1613–1618.

8 Hajra KM, Chen DY, Fearon ER. The SLUG zinc-finger protein represses E-cadherin in breast cancer. Cancer Res. 2002;62(6):1613–1618.

9 Hajra KM, Chen DY, Fearon ER. The SLUG zinc-finger protein represses E-cadherin in breast cancer. Cancer Res. 2002;62(6):1613–1618.

10 Nieto MA. The snail superfamily of zinc-finger transcription factors. Nat Rev Mol Cell Biol. 2002;3(3):155–166.

11 Bolós V, Peinado H, Pérez-Moreno MA, Fraga MF, Esteller M, Cano A. The transcription factor Slug represses E-cadherin expression and induces epithelial to mesenchymal transitions: a comparison with Snail and E47 repressors. J Cell Sci. 2003;116(Pt 3):499–511.

12 Hajra KM, Chen DY, Fearon ER. The SLUG zinc-finger protein represses E-cadherin in breast cancer. Cancer Res. 2002;62(6):1613–1618.

13 Han CB, Li F, Ma JT, Zou HW. Concordant KRAS mutations in primary and metastatic colorectal cancer tissue specimens: a meta-analysis and systematic review. Cancer Invest. 2012;30(10):741–747.

14 Watanabe T, Kobunai T, Yamamoto Y, Matsuda K, Ishihara S, Nozawa K, et al. Heterogeneity of KRAS status may explain the subset of discordant KRAS status between primary and metastatic colorectal cancer. Dis Colon Rectum. 2011;54(9):1170–1178.

15 Wang XQ, Li H, Van Putten V, Winn RA, Heasley LE, Nemenoff RA. Oncogenic K-Ras Regulates Proliferation and Cell Junctions in Lung Epithelial Cells through Induction of Cyclooxygenase-2 and Activation of Metalloproteinase-9. Mol Biol Cell. 2009;20(3):791–800.

16 Peinado H, Olmeda D, Cano A. Snail, Zeb and bHLH factors in tumour progression: an alliance against the epithelial phenotype? Nat Rev Cancer. 2007;7(6):415–428.

17 Dohadwala M, Yang SC, Luo J, Sharma S, Batra RK, Huang M, et al. Cyclooxygenase-2–Dependent Regulation of E-Cadherin: Prostaglandin E2 Induces Transcriptional Repressors ZEB1and Snail in Non–Small Cell Lung Cancer. Cancer Res. 2006;66(10):5338–5345.

18 Rebollo A, A CM. Ras Proteins: Recent Advances and New Functions. Blood. 1999;94(9):2971–2980.

19 Hanson JL, AnestV, Madrid JR, Baldwin AS. Oncoprotein Suppression of Tumor Necrosis Factor-induced NFκB Activation Is Independent of Raf-controlled Pathways. J Biol Chem. 2003;278(37):34910–34917.

20 Liss AS, Bose HR. Characterization of ATF2 in Rel/NFκBoncogenesis reveals its role in the regulation of Ras signaling. Small GTPases. 2011;2(2):89–94.

21 Finco TS, Westwick JK, Norris JL, Beg AA, Der CJ, Baldwin AS, Jr. Oncogenic Ha-Ras-induced Signaling Activates NF-kB Transcriptional Activity, Which Is Required for Cellular Transformation. J Biol Chem. 1997;272(39):24113–24116.

22 Bassères DS, Ebbs A, E Levantini, AS Baldwin. Requirement of the NF-κB Subunit p65/RelA for K-Ras–Induced Lung Tumorigenesis. Cancer Res. 2010; 70(9):3537–3546.

23 Arsura M, Mercurio F, Oliver AL, Thorgeirsson SS, Sonenshein GE. Role of the IκB Kinase Complex in Oncogenic Ras- and Raf-Mediated Transformation of Rat Liver Epithelial Cells. Mol Cell Biol. 2000;20(15):5381–5391.

24 Liu Y, Mayo MW, Nagji AS, Smith PW, Ramsey CS, Li D, et al. Phosphorylation of RelA/p65 promotes DNMT-1 recruitment to chromatin and represses transcription of the tumor metastasis suppressor gene BRMS1. Oncogene. 2012;31(9):1143–1154.

25 Hoesel B, Schmid JA. The complexity of NF-κB signaling in inflammation and cancer. Mol Cancer. 2013;12:86.

26 Xiao J, Duan X, Yin Q, Miao Z, Yu H, Chen C, et al. The inhibition of metastasis and growth of breast cancer by blocking the NFκB signaling pathway using bioreducible PEI-based/p65 shRNA complex nanoparticles. Biomaterials. 2013;34(21):5381–5390.

27 Huber MA, Azoitei N, Baumann B, Grünert S, Sommer A, Pehamberger H, et al. NF-κB is essential for epithelialmesenchymal transition and metastasis in a model of breast cancer progression. J Clin Invest. 2004;114(4):569–581.

28 Fraser DM, Sullivan FM, Thompson AM, McCowan C. Aspirin use and survival after the diagnosis of breast cancer: a population-based cohort study. Br J Cancer. 2014;111(3):623–627.

29 Holick CN, Michaud DS, Leitzmann MF, Willett WC, Giovannucci E. Aspirin use and lung cancer in men. Br J Cancer. 2003;89(9):1705–1708.

30 Dyke ALV, Cote ML, Prysak G, Claeys GB, Wenzlaff AS, Schwartz AG. Regular Adult Aspirin Use Decreases the Risk of Non-Small Cell Lung Cancer among Women. Cancer Epidemiol Biomarkers Prev. 2008;17(1):148–157.

31 Moysich KB, Menezes RJ, Ronsani A, Swede H, Reid ME, Cummings KM, et al. Regular aspirin use and lung cancer risk. BMC Cancer. 2002;2:31.

32 Wang HL, Lopategui J, Amin MB, Patterson SD. KRAS Mutation Testing in Human Cancers: The Pathologist’s Role in the Era of Personalized Medicine. Adv Anat Pathol. 2010;17(1):23–32.

33 Wang Y, Ngo VN, Marani M, Yang Y, Wright G, Staudt LM, et al. Critical role for transcriptional repressor Snail2 in transformation by oncogenic RAS in colorectal carcinoma cells. Oncogene. 2010;29(33):4658–4670.

34 Cho Y, Gorina S, Jeffrey PD, Pavletich NP. Crystal structure of a p53 tumor suppressor-DNA complex: understanding tumorigenic mutations. Science. 1994;265:346–355.

35 Adhikary A, Chakraborty S, Mazumdar M, Ghosh S, Mukherjee S, Manna A, et al. Inhibition of Epithelial to Mesenchymal Transition by E-cadherin Up-regulation via Repression of Slug Transcription and Inhibition of E-cadherin Degradation Dual Roleof Scaffold/Matrix Attachment Region-Binding Protein 1 (SMAR1) in Breast Cancer Cells. J Biol Chem. 2014;289(37):25431–25444.

36 Bolós V, Peinado H, Pérez-Moreno MA, Fraga MF, Esteller M, Cano A. The transcription factor Slug represses E-cadherin expression and induces epithelial to mesenchymal transitions: a comparison with Snail and E47 repressors. J Cell Sci. 2003;116:499-511.

37 Roberts PJ, Der CJ. Targeting the Raf-MEK-ERK mitogen-activated protein kinase cascade for the treatment of cancer. Oncogene. 2007;26(22):3291–3310.

38 Aplin AE, Stewart SA, Assoian RK, Juliano RL. Integrin-Mediated Adhesion Regulates ERK Nuclear Translocation and Phosphorylation of Elk-1. J Cell Biol. 2001;153(2):273–281.

39 Shin SY, Kim CG, Lim Y, Lee YH. The Ets Family Transcription Factor Elk-1 Regulates Induction of the Cell cycle Regulatory Gene p21^Waf1/Cip1^ and the Bax Gene in Sodium Arsenite-Exposed Human Keratinocyte HaCaT cells. J Biol Chem. 2011;286(30):26860–26872.

40 Yang SH, Yates PR, Whitmarsh AJ, Davis RJ, Sharrocks AD. The Elk-1 Ets-domain transcription factor contains a mitogen-activated protein kinase targeting motif. Mol Cell Biol. 1998;18(2):710–720.

41 Chang F, Steelman LS, Lee JT, Shelton JG, Navolanic PM, Blalock WL, et al. Signal transduction mediated by the Ras/Raf/MEK/ERK pathway from cytokine receptors to transcription factors: potential targeting for therapeutic intervention. Leukemia. 2003;17(7):1263–1293.

42 Mohanty S, Saha S, Hossain DMS, Adhikary A, Mukherjee S, Manna A, et al. ROS-PIASγccross talk channelizes ATM signaling from resistance to apoptosis during chemosensitization of resistant tumors. Cell Death Dis. 2014;5:e1021. doi:10.1038/cddis.2013.534.

43 Jo H, Zhang R, Zhang H, McKinsey TA, Shao J, Beauchamp RD, et al. NF-kB is required for H-ras oncogene induced abnormal cell proliferation and tumorigenesis. Oncogene. 2000;19(7):841–849.

44 Wu DW, Lee MC, Hsu NY, Wu TC, Wu JY, Wanget YC, et al. FHIT loss confers cisplatin resistance in lung cancer via the AKT/NF-κB/Slug-mediated PUMA reduction. Oncogene. 2015;34(19):2505-15.

45 Kopp E, Ghosh S. Inhibition of NF-kappa B by sodium salicylate and aspirin. Science. 1994;265(5174):956–959.

46 Yamamoto Y, Gaynor RB. Therapeutic potential of inhibition of the NF-κB pathway in the treatment of inflammation and cancer. J Clin Invest. 2001;107(2):135–142.

47 Thun MJ, Jacobs EJ, Patrono C. The role of aspirin in cancer prevention. Nat Rev Clin Oncol. 2012;9(5):259–267.

48 Norrish AE, Jackson RT, McRae CU. Non-steroidal anti-inflammatory drugs and prostate cancer progression. Int J Cancer. 1998;77(4):511–515.

49 Gilmore TD, Herscovitch M. Inhibitors of NFκB signaling: 785 and counting. Oncogene. 2006;25(51):6887–6899.

50 Rothwell PM, Wilson M, Price JF, Belch JF, Meade TW, MehtaZ. Effect of daily aspirin on risk of cancer metastasis: a study of incident cancers during randomised controlled trials. Lancet. 2012;379(9826):1591–1601.

51 Algra AM, Rothwell PM. Effects of regular aspirin on long-term cancer incidence and metastasis: a systematic comparison of evidence from observational studies versus randomised trials. Lancet Oncol. 2012;13(5):518–527.

52 Wynne S, Djakiew D. NSAID Inhibition of Prostate Cancer Cell Migration Is Mediated by Nag-1 Induction via the p38 MAPK-p75(NTR) Pathway. Mol Cancer Res. 2010;8(12):1656–1664.

53 Schrör, K. Clinical Applications of Aspirin, In: Acetylsalicylic Acid, Wiley-VCH Verlag GmbH & Co. KGaA, Weinheim, Germany. 2008.

54 Brady RR, Loveridge CJ, Dunlop MG, Stark LA. c-Src dependency of NSAID-induced effects on NF-kB-mediated apoptosis in colorectal cancer cells. Carcinogenesis. 2011;32(7):1069–1077.

55 Kalluri R, Weinberg RA. The basics of epithelial-mesenchymal transition. J Clin Invest. 2009;119(6):1420–1428.

56 Tsai JH, Yang J. Epithelial–mesenchymal plasticity in carcinoma metastasis. Genes Dev. 2013;27(20):2192–2206.

57 Lemieux E, Cagnol S, Beaudry K, Carrier J, Rivard N.^.^ Oncogenic KRAS signalling promotes the Wnt/β-catenin pathway through LRP6 in colorectal cancer. Oncogene. 2015;34(38):4914-27.

58 Shin S, Blenis J. ERK2/Fra1/ZEB pathway induces epithelial-tomesenchymal transition. Cell Cycle. 2010;9(13):2483–2484.

59 Li QJ, Yang SH, Maeda Y, Sladek FM, Sharrocks AD, Martins-Green M. MAP kinase phosphorylation-dependent activation of Elk–1 leads to activation of the co-activator p300. EMBO J. 2003;22(2):281–291.

60 Li QJ, Yang SH, Maeda Y, Sladek FM, Sharrocks AD, Martins-Green M. MAP kinase phosphorylation-dependent activation of Elk-1 leads to activation of the co-activator p300. EMBO J. 2003;22(2):281–291.

61 Li QJ, Yang SH, Maeda Y, Sladek FM, Sharrocks AD, Martins-Green M. MAP kinase phosphorylation-dependent activation of Elk-1 leads to activation of the co-activator p300. EMBO J. 2003;22(2):281–291.

62 Bassères DS, Ebbs A, E Levantini, AS Baldwin. Requirement of the NF-κB Subunit p65/RelA for K-Ras–Induced Lung Tumorigenesis. Cancer Res. 2010; 70(9):3537–3546. doi: 10.1158/0008%E2%80%935472.CAN-09%E2%80%934290.

63 Bassères DS, Ebbs A, E Levantini, AS Baldwin. Requirement of the NF-κB Subunit p65/RelA for K-Ras–Induced Lung Tumorigenesis. Cancer Res. 2010; 70(9):3537–3546. doi: 10.1158/0008%E2%80%935472.CAN-09%E2%80%934290.

64 Arsura M, Mercurio F, Oliver AL, Thorgeirsson SS, Sonenshein GE. Role of the IκB Kinase Complex in Oncogenic Ras- and Raf-Mediated Transformation of Rat Liver Epithelial Cells. Mol Cell Biol. 2000;20(15):5381–5391.

65 Mohanty S, Saha S, Hossain DMS, Adhikary A, Mukherjee S, Manna A, et al. ROS-PIASγccross talk channelizes ATM signaling from resistance to apoptosis during chemosensitization of resistant tumors. Cell Death Dis. 2014;5:e1021. doi:10.1038/cddis.2013.534.

66 Wang Y, Yue B, Yu X, Wang Z, Wang M. SLUG is activated by nuclear factor kappa B and confers human alveolar epithelial A549 cells resistance to tumor necrosis factor-alpha induced apoptosis. World J Surg Oncol. 2013 Jan 22;11:12. doi: 10.1186/1477%E2%80%937819%E2%80%9311-12.

67 Brandl M, Seidler B, Haller F, Adamski J, Schmid RM, Saur D, et al. IKKαcontrols canonical TGFβ–SMAD signaling to regulate genes expressing SNAIL and SLUG during EMT in Panc1 cells. J Cell Sci. 2010;123(Pt 24):4231–4239.

68 Yotsui T, Yasuda O, Kawamoto H, Higuchi M, Chihara Y, Umemoto E, et al. Aspirin prevents adhesion of T lymphoblasts to vascular smooth muscle cells. FEBS Lett. 2007;581(3):427–432.

69 Brady RR, Loveridge CJ, Dunlop MG, Stark LA. c-Src dependency of NSAID-induced effects on NF-kB-mediated apoptosis in colorectal cancer cells. Carcinogenesis. 2011;32(7):1069–1077.

70 Helbig G, Christopherson KW2nd, Bhat-Nakshatri P, Kumar S, Kishimoto H, Miller KD, et al. NFkB Promotes Breast Cancer Cell Migration and Metastasis by Inducing the Expression of the Chemokine Receptor CXCR4. J Biol Chem. 2003;278(24):21631–21638.

71 Sobolewski C, Cerella C, Dicato M, Ghibelli L, Diederich M. The Role of Cyclooxygenase-2 in Cell Proliferation and Cell Death in Human Malignancies. Int J Cell Biol. 2010;2010:215158.

72 Chakraborty S, Mazumdar M, Mukherjee S, Bhattacharjee P, Adhikary A, Manna A, et al. Restoration of p53/miR-34a regulatory axis decreases survival advantage and ensures Bax-dependent apoptosis of non-small cell lung carcinoma cells. FEBS Lett. 2014;588(4):549–559.

73 Adhikary A, Chakraborty S, Mazumdar M, Ghosh S, Mukherjee S, Manna A, et al. Inhibition of Epithelial to Mesenchymal Transition by E-cadherin Up-regulation via Repression of Slug Transcription and Inhibition of E-cadherin Degradation Dual Role of Scaffold/Matrix Attachment Region-Binding Protein1 (SMAR1) in Breast Cancer Cells. J Biol Chem. 2014;289(37):25431–25444.

74 Saha B, Adhikary A, Ray P, Saha S, Chakraborty S, Mohanty S, et al. Restoration of tumor suppressor p53 by differentially regulating pro- and anti-p53 networks in HPV-18-infected cervical cancer cells. Oncogene. 2012;31(2):173–186.

75 Mukherjee S, Mazumdar M, Chakraborty S, Manna A, Saha S, Khan P, et al. Curcumin inhibits breast cancer stem cell migration by amplifying the E-cadherin/β-catenin negative feedback loop. Stem Cell Res Ther. 2014;5(5):116.

76 Lahiry L, Saha B, Chakraborty J, Adhikary A, Mohanty S, Hossain DMS, et al. Theaflavins target Fas/caspase-8 and Akt/pBad pathways to induce apoptosis in p53-mutated human breast cancer cells. Carcinogenesis. 2010;31(2):259–268.

77 Hossain DMS, Panda AK, Manna A, Mohanty S, Bhattacharjee P, Bhattacharyya S, et al. FoxP3 Acts as a Cotranscription Factor with STAT3 in Tumor-Induced Regulatory T Cells. Immunity. 2013;39(6):1057–1069.

78 Mazumdar M, Adhikary A, Chakraborty S, Mukherjee S, Manna A, Saha S, et al. Targeting RET to induce medullary thyroid cancer cell apoptosis: an antagonistic interplay between PI3K/Akt and p38MAPK/caspase-8 pathways. Apoptosis. 2013; 18(5):589–604.

79 Saha S, MukherjeeS, MazumdarM, MannaA, KhanP, AdhikaryA, et al. Mithramycin A sensitizes therapy-resistant breast cancer stem cells toward genotoxic drug doxorubicin. Transl Res. 2015;165(5):558–577.

80 Chakraborty J, Banerjee S, Ray P, DMS Hossain, S Bhattacharyya, Adhikary A, et al. Gain of Cellular Adaptation Due to Prolonged p53 Impairment Leads to Functional Switchover from p53 to p73 during DNA Damage in Acute Myeloid Leukemia Cells. J Biol Chem. 2010;285(43):33104–33112.

81 Chakraborty S, Das K, Saha S, Mazumdar M, Manna A, Chakraborty S, et al. Nuclear Matrix Protein SMAR1 Represses c-Fos-mediated HPV18 E6 Transcription through Alteration of Chromatin Histone Deacetylation. J Biol Chem. 2014;289(42):29074–29085.
