# Supplementary figures and images for: Aspirin inhibits epithelial-to-mesenchymal transition and migration of oncogenic K-ras-expressing non-small cell lung carcinoma cells by down-regulating E-cadherin repressor Slug
Source: BMC Cancer. 2016 Jan 26;16:39. doi: 10.1186/s12885-016-2078-7 (PMC4727308; doi:10.1186/s12885-016-2078-7)

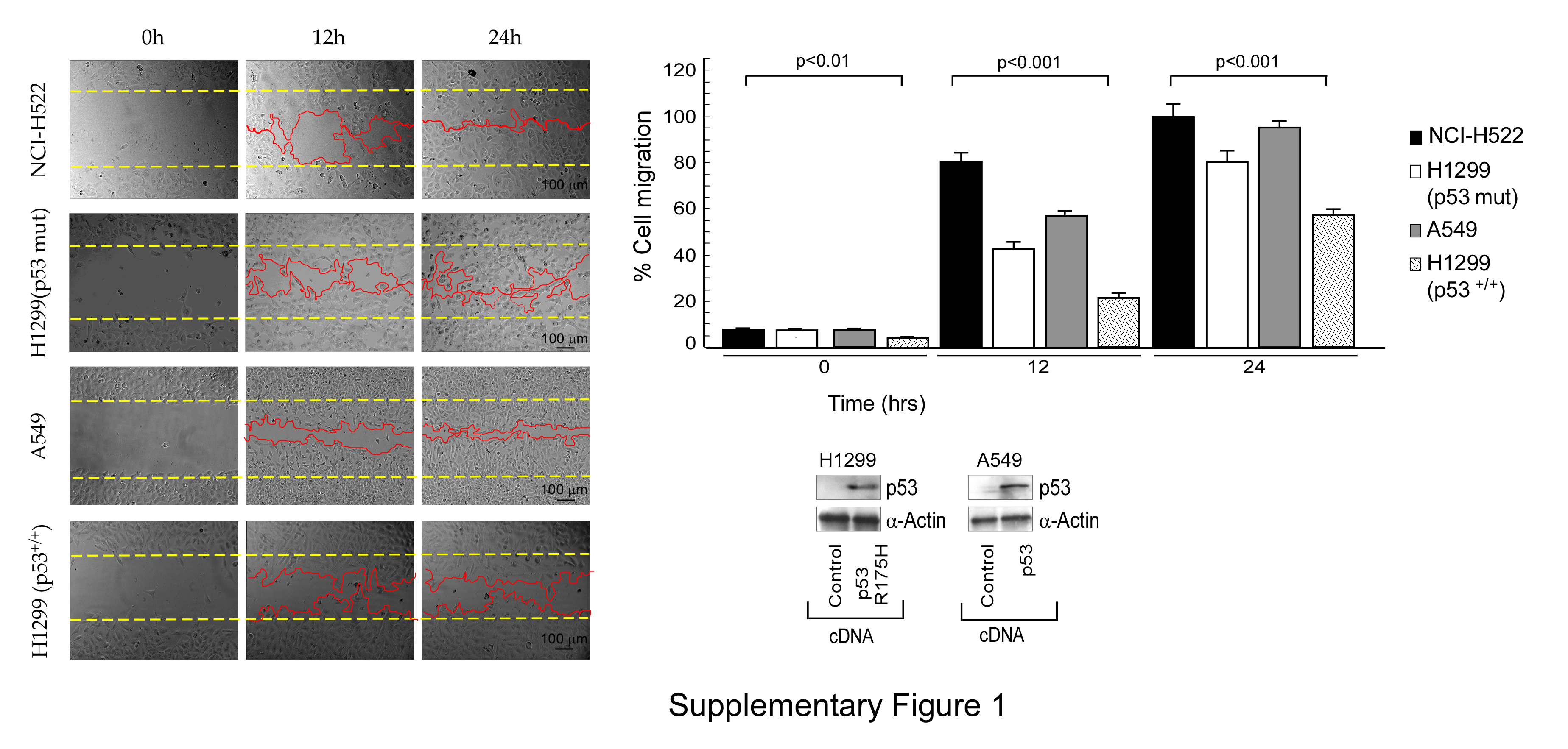

Supplement: Additional file 1: Figure S1. — p53 mutation exerts increased migratory effect in combination with oncogenic K-ras-expressing system on NSCLC cells migration. Phase contrast images (left panels) depicting migration of NCI-H522 cells (oncogenic K-ras/mutant p53), mutant p53-reconstituted H1299 cells (wild type K-ras/mutated p53), A549 cells (oncogenic K-ras/wild type p53), and wild type p53 reconstituted H1299 cells (wild type K-ras/wild type p53). Graphical representation of percent cell migration in wound healing assay (right panel) with inset showing immunoblot analysis for the transfection efficiency of p53 - R175H clone in H1299 cells and p53 - cDNA in H1299 cells. Scale bar: 100 μm. (TIFF 2457 kb) [file 12885_2016_2078_MOESM1_ESM.tiff]
